# Revealing the hierarchy of processes and time-scales that control the tropic response of shoots to gravi-stimulations

**DOI:** 10.1093/jxb/erz027

**Published:** 2019-03-27

**Authors:** Hugo Chauvet, Bruno Moulia, Valérie Legué, Yoël Forterre, Olivier Pouliquen

**Affiliations:** 1 Université Clermont Auvergne, INRA, PIAF, Clermont-Ferrand, France; 2 Aix Marseille University, CNRS, IUSTI, Marseille, France

**Keywords:** Gravitropism, gravity, kinematics, memory, modeling

## Abstract

Gravity is a major abiotic cue for plant growth. However, little is known about the responses of plants to various patterns of gravi-stimulation, with apparent contradictions being observed between the dose-like responses recorded under transient stimuli in microgravity environments and the responses under steady-state inclinations recorded on earth. Of particular importance is how the gravitropic response of an organ is affected by the temporal dynamics of downstream processes in the signalling pathway, such as statolith motion in statocytes or the redistribution of auxin transporters. Here, we used a combination of experiments on the whole-plant scale and live-cell imaging techniques on wheat coleoptiles in centrifuge devices to investigate both the kinematics of shoot-bending induced by transient inclination, and the motion of the statoliths in response to cell inclination. Unlike previous observations in microgravity, the response of shoots to transient inclinations appears to be independent of the level of gravity, with a response time much longer than the duration of statolith sedimentation. This reveals the existence of a memory process in the gravitropic signalling pathway, independent of statolith dynamics. By combining this memory process with statolith motion, a mathematical model is built that unifies the different laws found in the literature and that predicts the early bending response of shoots to arbitrary gravi-stimulations.

## Teaser

Experiments on shoot gravitropism at the plant and cell scale reveal the existence of a memory process and provide a unifying framework for predicting the bending response of shoots to arbitrary gravi-stimulations.

## Introduction

Plants have developed the ability to sense their inclination relative to the local vertical orientation as defined by the vector of gravitational acceleration **g**, and to adjust their shape accordingly, a process called gravitropism. The main gravity sensor of plants is located in specialized cells called statocytes, in which starch-filled plastids (statoliths) are found (Moulia and Fournier, 2009; Morita, 2010). Statoliths are denser than the surrounding cellular fluid and sediment at the bottom of the cell. When the plant is inclined, statoliths flow along the direction of gravity and trigger signalling processes, which induce the bending of the organ. A widely accepted step in this process is the creation of an auxin asymmetry between the upper and the lower part of the organ, which mediates differential growth and results in the bending of the plant. This asymmetric distribution, which is observed for shoots and roots (Harrison and Masson, 2008; Rakusová *et al.*, 2011; Brunoud *et al.*, 2012; Band *et al.*, 2012), seems to be driven by the redistribution of auxin transporters (PIN proteins) along the statocyte membranes (Friml *et al.*, 2002; Rakusová *et al.*, 2011). However, the different steps and temporal sequences of the gravitropic signalling pathway from the initial intracellular perception by the statoliths to the growth-induced bending of the plant remain largely unknown (Su *et al.*, 2017).

One way to address these questions involves investigating the macroscopic bending kinematics of the plant in response to different temporal and intensity patterns of gravi-stimulation (Hart, 1992; Moulia and Fournier, 2009). So far two kinds of experiments have been reported in the literature, corresponding to two different stimuli. The first involves permanently inclining the base of the plant at different angles relative to gravity. The response, defined as the bending velocity, is found to vary with the sine of the initial inclination of the plant shoot, *θ*_init_, (for reviews see Sachs, 1887; Larsen, 1969; Iino *et al.*, 1996; (Moulia and Fournier, 2009; Pouliquen *et al.*, 2017). This relationship, known as the ‘sine law’, is at the origin of the ‘starch-statolith weight model’, which proposes that the sensor acts as a force sensor that is sensitive to the pressure exerted by weight of the statoliths on the lateral membrane of the statocyte (Perbal and Perbal, 1976; Galland, 2002; Leitz *et al.*, 2009). In a recent study, Chauvet *et al.* (2016) tested this hypothesis using centrifuge experiments to disentangle the effect of the direction (i.e. the angle between the initial inclination of the plant shoot and the gravity vector) and the intensity of the gravity vector on the plant shoot response. They showed that the plant response is proportional to sin(*θ*_init_) and not to *g*_eff_ × sin(*θ*_init_). This insensitivity to the effective gravity, *g*_eff_, reported for land angiosperms has also been observed for characean green algae (Limbach, 2005). The gravity sensor in plants thus works as a clinometer and not as a force sensor, thereby falsifying not only the ‘statolith pressure hypothesis’ but also the alternative ‘protoplast pressure hypothesis’ (for a more complete argument see Pouliquen *et al.*, 2017). These observations support an alternative ‘position sensor hypothesis’, in which the relevant parameter triggering the signal pathway is the position of the statoliths within the statocytes (Strohm *et al.*, 2014; Pouliquen *et al.*, 2017).

The second type of macroscopic experiments have focused on the establishment of a ‘dose–response’ by applying transient stimuli. Experiments have been carried out either in microgravity conditions (Perbal and Driss-Ecole, 1994; Brown *et al.*, 1995; Driss-Ecole *et al.*, 2008), where the plant is transiently exposed to different values of *g*_eff_ for a time ∆*t* using a centrifuge before gravity is switched again to zero, or in ground-based experiments using clinostats to compensate for the effects of Earth gravity on the statolith sedimentation and hence to suppress gravisensing (Larsen, 1957; Shen-Miller, 1970; Caspar and Pickard, 1989; Kiss *et al.*, 1989; Galland *et al.*, 2004). In all these experiments, the gravitropic response is usually quantified by a ‘dose–response’ curve, where the dose refers to the amount of the stimulus perceived by the sensor during the exposure time. For gravisensing, the dose is defined as *g*_eff_ × ∆*t* (Hart, 1992; Perbal *et al.*, 2002), although the quantification method of the response has differed between studies. The major purpose of these dose–response studies has been the determination of a sensitivity threshold, i.e. the minimum dose required to produce a response (Brown *et al.*, 1995; Perbal *et al.*, 2002). This is why they have been conducted under a range of low-gravity intensities.

This body of experimental work is difficult to amalgamate into a unified framework. Indeed, the observation of a ‘dose–response’ showing a response proportional to the intensity of gravity, *g*_eff_, in transient inclination experiments is in apparent contradiction to the ‘sine law’ obtained for a permanent inclination, with the latter showing that the response does not depend on gravity. Within the ‘position sensor hypothesis’, Pouliquen *et al.* (2017) have proposed that these two response laws could actually represent two different regimes of the same underlying process, depending on the duration of the stimulation compared to an intrinsic time-scale of the signalling pathway. They proposed that this intrinsic time-scale could be determined by the time taken by the statoliths to move in the statocytes in response to a gravitropic stimulus. During a permanent inclination, the final position of the statoliths is the same whatever the intensity of the gravity and only depends on the angle of inclination, which could explain why *g*_eff_ does not appear in the ‘sine law’. However, the amount of time necessary for statoliths to move and reach their final position is expected to depend on the intensity of *g*_eff_. If the duration of the stimulus, ∆*t*, is too short, statoliths may not have time to reach their final equilibrium position; the response is then expected to be a function of the displacement of the statoliths, which should depend on both ∆*t* and *g*_eff_.

The objective of the present study was to test the hypothesis of a crucial role of the time-scale for collective statolith motion by studying on the same system (wheat coleoptiles) both the tropic response of the plants and the motion of the statoliths when the plant is submitted to permanent or transient stimuli. The comparison between the observations at the two scales revealed a more complex scenario than initially anticipated, in which the gravitropic response is controlled by a whole hierarchy of time-scales, and not only by the avalanche time of the statoliths. Based on these observations, we have developed a mathematical model that extends previous kinematic models for plant gravitropism (Bastien *et al.*, 2013, 2014; Dumais, 2013) to unsteady stimuli. This model enables the shoot responses to arbitrary gravi-stimulation to be predicted and unified within a single framework.

## Materials and methods

### Plant material and culture conditions

Seeds of wheat (*Triticum aestivum* cv Demeter) were used for all experiments. For experiments at the plant scale, seeds were each individually glued at the top of one side of a small plastic box (35×25×10 mm). The boxes were open at the top and filled with cotton wool. The germ of the seed was pointing down toward the cotton wool to ensure an initial straight coleoptile. For experiments at the cell scale, around 30 seeds were dispersed in a plastic container (323×63 mm) with random orientation. In both sets of experiments, the seeds were placed in a humidification chamber located in a dark room with controlled temperature (24 °C). Experiments were carried out when the coleoptiles reach a size of ~1.5–2.5 cm (about 4 d after germination).

It should be noted that at this stage the cell division is over. Reports in the literature indicate that cell division ceases very early when the coleoptile is ~3 d old and with a size that ranges from 9 mm (Lu *et al.*, 2006) to 17 mm (Wright, 1961). Therefore, in all our experiments, the growth and differential growth involved only cell expansion. Bastien *et al.* (2013, 2014) also showed that at this stage the distribution of the relative elemental growth rate along the growth zone of the coleoptile is steady, so that both the elongation rate and the size of the elongation zone are steady.

### Experimental set-up at the plant scale: transient inclination at different gravity levels

**Fig. 1.**
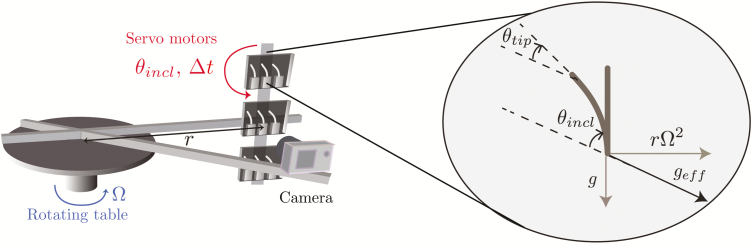
Experimental set-up used to study the responses of wheat coleoptiles subjected to transient inclinations for different gravity intensities, *g*_eff_. Three small chambers were fixed to a rotating table and controlled by servomotors to induce a transient inclination at an angle *θ*_incl_ from the direction of the effective gravity.

The experimental set-up used to study the response to transient stimuli is illustrated in Fig. 1. Three small chambers (110×110×30 mm) were fixed on a rotating table (Shimpo RK-3E, Shimpo Kyoto Japan). Each chamber contained three coleoptiles and was attached to a servomotor to precisely control their orientation. The whole set-up was placed inside a dark room at a temperature of 24 °C. The rotating table was put in rotation at Ω=1 rotations s^–1^ to create centrifugal acceleration equivalent to three times the Earth’s gravity (i.e. *g*_eff_=3*g*) at the location of the chambers, or it remained static for experiments at *g*_eff_=1*g*. A camera (Nikon D200) was fixed in front of the three chambers and was synchronized with a flash. The camera shutter and the servomotors controlling the inclination of the chambers were monitored using an open-source microcontroller (Arduino; https://www.arduino.cc/). Before experiments, three wheat coleoptiles in their small boxes were placed in each chamber (Fig. 1). A time sequence of inclination angles was then uploaded to the microcontroller memory (see Results). The sequence started with a resting period (~6 h) when the coleoptiles were aligned with the effective gravity (vertical for *g*_eff_=1*g* but inclined when *g*_eff_=3*g*), followed by a stimulation period ∆*t* when an inclination angle *θ*_incl_ was imposed, and finally the coleoptiles were re-aligned with the direction of the effective gravity. Time-lapse images are taken every hour during the resting period and the frequency was increased by the microcontroller to one image every 5 min once the stimulation had started. For each ∆*t*, the responses of several coleoptiles (between 9–27) were analysed.

### Live-cell imaging of the motion of statolith at different gravity levels

**Fig. 2.**
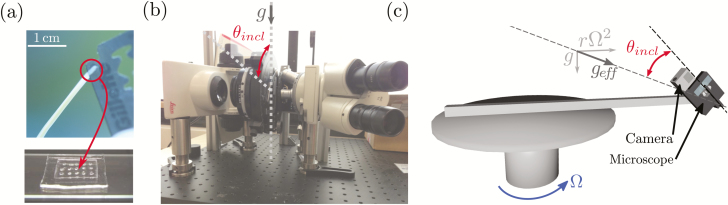
Experimental set-ups used to observe *in vivo* statolith movements at 1*g* and 3*g* in wheat coleoptiles. (a) Thin longitudinal slices of the apex from coleoptiles were cut and fixed in cavities in a layer of polydimethylsiloxane filled with D-sobitol. (b) A bright-field microscope was inclined horizontally such that the stage rotated in a vertical plane. Time-lapse images of the statoliths were taken after an inclination *θ*_incl_ was applied to the sample. (c) To study the statolith dynamics at 3*g*, a compact microscope was mounted on a servomotor, which was fixed on a rotating table. The servomotor controlled the inclination *θ*_incl_ of the microscope relative to *g*_eff_.

To observe the dynamics of the statoliths, a small longitudinal slice (a few millimetres long) was manually cut at the apex of the coleoptile using a razor blade (Fig. 2a). The slice was done at the sharper edge of the coleoptile where the vascular tissue is located and along which the statocytes are positioned. Up to four slices were then enclosed in a small cavity made in polydimethylsiloxane (PDMS) fixed to a microscope slide (Fig. 2a). The cavity was filled with a solution of D-sorbitol (0.2 mol l^–1^) and closed with a cover slide, which ensured an *in vivo* state for several hours. For experiments at *g*_eff_=1*g*, a bright-field microscope (Leica DM 2500P) equipped with a camera (Nikon D300) was tilted on its side so that the stage plane aligned with the direction of gravity (Fig. 2b). When the stage rotated the statoliths moved in the focal plane of the microscope and time-lapse images were taken to follow their motion. For experiments at *g*_eff_=3*g*, a very compact microscope (NM1 Newton microscope) equipped with a small camera (GoPro Hero 4) was used and placed on the same rotating table as described above (Fig. 2c). The inclination of the microscope was controlled by a servomotor through the Arduino microcontroler. In both set-ups, the procedure to analyse the statolith dynamics was the same. The sample was kept fixed for several minutes with the cells aligned with the direction of gravity (vertical at 1*g* and aligned with *g*_eff_ at 3*g* on the rotating table) in order to let the statoliths sediment at the bottom of the cell and form a pile. Observing sedimentation is an efficient way to select cells that act as statocyte cells, by selecting only the ones containing statoliths denser than the intercellular medium. A sudden inclination was then applied using the rotating stage and acquisition of data commenced.

### Image analysis

**Fig. 3.**
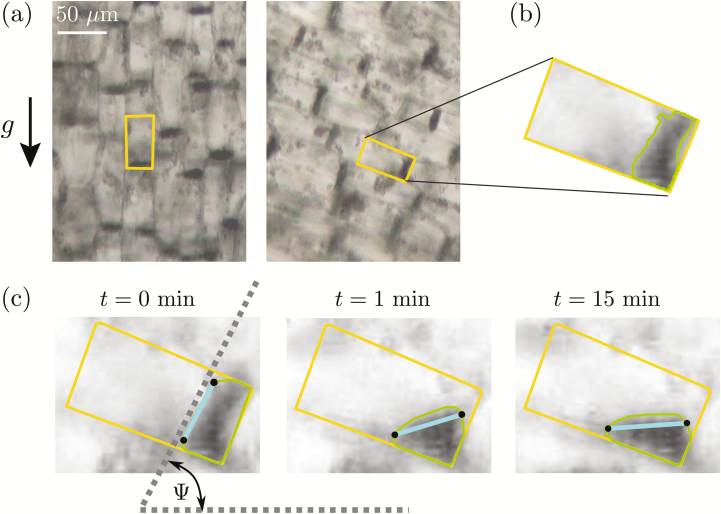
Image analysis of statolith movements in response to cell inclination in wheat coleoptiles. (a) Bright-field images with several statocytes, before and after a rotation at an angle *θ*_incl_ = 70°. The rectangles show the outlines of individual statocytes. (b) The outline of the pile formed by the statoliths was extracted using a contour-finding algorithm based on a gray-scale threshold level. (c) The free surface (thick line between points) was determined and the time-response of its angle *Ψ*(*t*) relative to horizontal was recorded.

In the plant-scale experiments, the spatio-temporal responses of the coleoptiles were measured using the same software that was developed and described by Chauvet *et al.* (2016). The topological skeleton of coleoptile was first extracted and then analysed to compute the response of the coleoptile length over time, *L*(*t*) and of the tip angle, *θ*_tip_(*t*) (Fig. 1), defined as the spatial mean inclination of the skeleton at the tip over a length equal to two mean coleoptile diameters. This inclination was measured relative to the direction of the local gravity (vertical at *g*_eff_=1*g* and inclined at *g*_eff_=3*g*). For experiments at the cell scale, the bright-field microscopy images are analysed using a specific Python script and the scikit-image library (van der Walt *et al.*, 2014). The outlines of the statocyte cells were first delineated manually (Fig. 3a). For each selected cell, the image was then cropped along the cell outline and a local minimum filter with a size of about one statolith diameter was applied inside the cell such that the pile of sedimented statoliths appeared as a uniform black area. This area was finally extracted using a contour-finding algorithm based on a gray-scale threshold level (Fig. 3b). To quantify the motion of the statolith pile inside the cell, the angle of the pile free surface relative to horizontal, *Ψ*, was computed (Fig. 3c).

### Theoretical model

We developed a mathematical model to describe the spatio-temporal responses of a shoot subjected to an arbitrary gravi-stimulation. We have followed previous kinematic models, and first describe the relationship between the rate of change of the curvature, *C*, of the shoot and the differential elongation rate $\Delta \dot{\varepsilon }$ between the top and the bottom faces of the shoot in response to the gravi-stimulation:

$$\begin{array}{l} R\frac{\partial C\left(s,t\right)}{\partial t}=\frac{1}{\tau_{growth}}\times \frac{\Delta \dot{\epsilon }}{2\dot{\epsilon }} \end{array}$$ (1)

where *R* is the radius of the shoot and is assumed uniform, and *τ*_growth_ is the growth time equal to the inverse of the mean elongation rate $\dot{\varepsilon }$ (i.e. $\tau_{\text{growth}}=1/\dot{\varepsilon }$) of the stem (Moulia and Fournier, 2009). This equation is derived from the simplification of a more general model accounting for growth-induced motions of the cell wall materials along the tropic growth response (for the complete derivation see Bastien *et al.*, 2014). This simplification holds when (i) the time-scale of the tropic bending is small compared to the time-scale of the cell elongation-induced displacement, and (ii) the size of the growth zone is steady and the growth zone spatially coincides with the tropic response zone. In our experiments, the bending of the coleoptile was fast (~1 h) compared to cell elongation growth (*τ*_growth_ ~17 h), and the growth zone of the coleoptile coincides with the tropic bending zone.

In a series of studies Bastien *et al.* (2013, 2014) assumed that the relative differential growth was controlled by the sum of a gravitropic signal *G*_s_ and of a proprioception signal *P*_r_:

$$\begin{array}{l} \frac{\;\Delta \;\dot{\epsilon }}{2\dot{\epsilon }}=G_{s}+P_{r} \end{array}$$ (2)

with the gravitropic signal being proportional to the sine of the inclination *A* of the stem relative to the vertical, $G_{\text{s}}=-\underline{\beta }\sin\;(A)$ where $\underline{\beta }$ is the gravitropic sensitivity, and the proprioception being proportional to the curvature $C,P_{r}=-\bar{\gamma }\text{CR}$, where $\tilde{\gamma }$ is the proprioceptive sensitivity. Bastien *et al*.’s model (AC model) described the spatio-temporal kinematics of shoots subjected to permanent inclinations and the possible overshoot from the vertical, which is controlled by the relative importance of the gravitropic sensitivity and of the proprioception. However, the AC model cannot capture the dose–response under transient inclination as applied in our study, as the gravitropic signal is assumed to respond instantaneously to the shoot angle *A*. Here, we extend this model using the ‘position sensor hypothesis’ (Pouliquen *et al.*, 2017), which stipulates that the relevant parameter controlling the gravitropic signal is the position of the statolith pile within the cell. In addition, we assume that the position of the statoliths is not instantaneously transcribed into differential growth, but that the gravitropic signal results from an integration process of the statolith position with a memory time and a delay. The new model, termed A-SD-M (for angle–statolith dynamic–memory), thus contains two additional ingredients, namely the displacement of the statoliths and the integration process, which are described below.

### Statolith avalanche

**Fig. 4.**
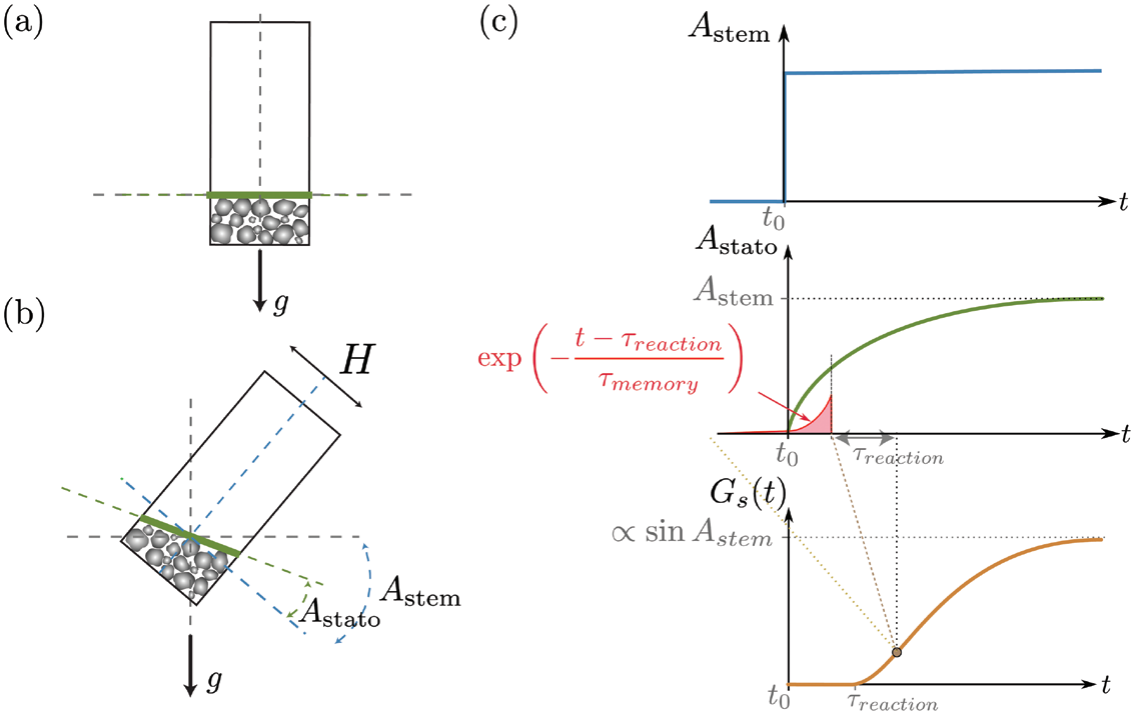
Description of the theoretical model for responses of wheat coleoptiles subjected to transient inclination. (a) The initial state when the statocyte is aligned with gravity: the statoliths have sedimented and form a pile. The free surface of the pile (thick line) is perpendicular to the long axis of the statocyte (*A*_stato_ = 0). (b) When the statocyte is inclined at an angle *A*_stem_, the statolith pile flows and the angle of the surface increases so that *A*_stato_ tends to *A*_stem_. (c) Typical responses of the variables of the model. When the stem inclination suddenly changes (top graph), the pile angle increases and tends to *A*_stato_ = *A*_stem_ (middle graph). The gravitropic signal *G*_s_ starts to increase after a time delay *τ*_reaction_ (bottom) and results from the integration of the statolith angle *A*_stato_ over a memory time *τ*_memory_ with an exponential decay as indicated in the middle graph.

When the stem is inclined (Fig. 4b), the pile formed by the dense statoliths at the bottom of the cell moves like an avalanche of grains and the dynamics are characterized by the evolution over time of the pile inclination *A*_stato_(*t*) relative to the cell inclination *A*_stem_. In contrast to a classical granular medium, we assume that the pile after the avalanche returns to the horizontal and presents a free surface perpendicular to the gravity vector. This absence of a repose angle is suggested by experimental observations (Bérut *et al.*, 2018) and is discussed in Pouliquen *et al.* (2017). The dynamics are described by a viscous relaxation of the pile towards the horizontal position, leading to the following equation:

$$\begin{array}{l} \frac{d\;A_{stato}}{dt}=-\frac{1}{\tau_{aval}}\sin\left(A_{stato}-A_{stem}\right) \end{array}$$ (3)

*τ*_aval_ is the characteristic duration of the avalanche and is quantitatively estimated from experimental observations. A typical response curve of the free surface angle *A*_stato_ following a sudden inclination of the stem is shown in Fig. 4c. A theoretical expression of *τ*_aval_ is derived by assuming that the dynamics are controlled by the balance between the gravitational driving force *Mg*_eff_ sin(*A*_stato_ –*A*_stem_), where *M* is the buoyancy-free mass of the statolith pile (proportional to the density difference ∆*ρ* between the starch and the water), and a viscous force proportional to *ηH*^2^.d(*A*_stato_)/d*t* (Guyon *et al.*, 2015), where *η* is the viscosity of the intracellular fluid and *H* is the typical thickness of the pile. The scaling of the avalanche duration is then given by:

$$\begin{array}{l} \tau_{aval}=\alpha \frac{\eta }{\;\Delta \rho Hg_{eff}\;} \end{array}$$ (4)

Where α is a pre-factor that may depend on other dimensionless parameters such as the aspect ratio of the statolith pile or the number of statoliths. Note that the avalanche duration *τ*_aval_ is inversely proportional to the gravity intensity *g*_eff_, meaning that it increases when gravity decreases.

### The gravitropic signal

We next assume that the gravitropic signal *G*_s_ controlling the differential growth on the two faces of the inclined stem is linked to the position of the statoliths, *A*_stato_, by an integrative process. Specifically, we assume that the gravitropic signal at time *t* is given by the integral of the sine of the angle of the statoliths *A*_stato_(*t*´<*t*) over a memory time *τ*_memory_ after a global time delay *τ*_reaction_. The decay function associated with the memory process, i.e. the kernel of the integral, is chosen to be exponential for the sake of simplicity. Mathematically, the expression of the gravitropic signal is written as:

$$\begin{array}{l} G_{s}\left(t\right)=\beta \frac{1}{\tau_{memory}}\int_{-\infty }^{{t-\tau }_{reaction}}\exp\left(-\frac{t-\tau_{reaction}-t^{\prime }}{\tau_{memory}}\right)\sin\left[A_{stato}\left(t^{\prime }\right)\right]dt^{\prime } \end{array}$$ (5)

where *β* is a constant. After a derivation for time, eqn (5) can be written as:

$$\begin{array}{l} \frac{\partial G_{s}\left(t\right)}{\partial t}=\frac{1}{\tau_{memory}}\left\{\beta \sin\left[A_{stato}\left(t-\tau_{reaction}\right)\right]-G_{s}\left(t\right)\right\} \end{array}$$ (6)

The typical response of *G*_s_ following a sudden inclination is shown in Fig. 4c.

### The final A-SD-M model

To finalize the model, we assume that eqns (1), (2), (3), and (6) apply at each location of the stem indexed by the curvilinear abscissa *s*, the local curvature being related to the stem angle by *C*(*s*,*t*)=∂*A*_stem_(*s*,*t*)/∂*s* (Moulia and Fournier, 2009; Bastien *et al.*, 2013). The response of a stem to an arbitrary time-dependent inclination applied at its base *A*_stem_(0,*t*) is then given by the following set of four differential equations:

$$\begin{array}{l} R\frac{\partial C\left(s,t\right)}{\partial t}=-\frac{1}{\tau_{growth}}G_{s}\left(s,t\right) \end{array}$$ (7)

$$\begin{array}{l} \frac{\partial G_{s}\left(s,t\right)}{\partial t}=\frac{1}{\tau_{memory}}\left\{\beta \sin\left[A_{stato}\left(s,t-\tau_{reaction}\right)\right]-G_{s}\left(s,t\right)\right\} \end{array}$$ (8)

$$\begin{array}{l} \frac{\partial A_{stato}\left(s,t\right)}{\partial t}=-\frac{1}{\tau_{aval}}\sin\left[A_{stato}\left(s,t\right)-A_{stem}\left(s,t\right)\right] \end{array}$$ (9)

$$\begin{array}{l} A_{stem}\left(s,t\right)=\int_{0}^{s}C\left(s,t\right)ds+A_{stem}\left(0,t\right) \end{array}$$ (10)

where the proprioception term is neglected, as we are interested only in describing situations where curvatures are small. This set of equations was solved numerically using a Python script based on a finite difference scheme.

### Calibration

In order to quantitatively compared the predictions of the model with the experimental measurements, the parameters needed to be calibrated. Some were directly obtained from the experimental measurements. The elongation rate, and thus the growth duration *τ*_growth_, and the radius *R* were measured in the experiments and the following values were used: *τ*_growth_=1200 min (ε = 8.4×10^–4^ min^–1^), and *R*=0.8 mm. The avalanche duration *τ*_aval_ was obtained by fitting the observed experimental response of the angle of the statolith piles with an exponential, giving *τ*_aval_=1.04 min for *g*_eff_=1*g*. The reaction time *τ*_reaction_ was given by the minimum time recorded before a response was observed, which was found to be 13 min. Finally, the parameter *β* and the integration time *τ*_memory_ were chosen equal to 0.8 and 13 min, respectively, to correctly fit the dose–response curves.

### The different regimes predicted by the A-SD-M model for the gravitropic response to transient inclinations at various values of *g*_eff_

In this study, we were interested in the response of a coleoptile inclined over a duration ∆*t* at an angle *θ*_incl_ before being returned to the vertical. Within certain limits, predictions can be made from the theoretical model described above, and these are discussed below.

To measure the response from data obtained from our experiments, we used the gravitropic sensitivity $\tilde{\beta }$ (Bastien *et al.*, 2014; Chauvet *et al.*, 2016), defined as the dimensionless speed of variation of the tip angle:

$$\begin{array}{l} \tilde{\beta }=\frac{R}{\sin\left(\theta_{incl}\right)}\frac{d\theta_{tip}/dt}{\left\langle dL/dt\right\rangle } \end{array}$$ (11)

where d*θ*_tip_/d*t* is the maximum slope during the first increase phase of the tip angle, <d*L*/d*t>* is the averaged growth velocity of the coleoptile, and *L* and *R* are the length and the radius of the coleoptile, respectively. $\tilde{\beta }$ has been shown to be independent of the angle of inclination, of the elongation growth rate, and of the size of the organ (Bastien *et al.*, 2013, 2014). We used the A-SD-M model to derive asymptotic solutions to predict how $\tilde{\beta }$ depends on the duration of the inclination ∆*t* and on the parameters of the model. According to A-SD-M, the reaction time *τ*_reaction_, the memory time *τ*_memory_, and the growth time *τ*_growth_ were constant and independent of the experimental conditions, with *τ*_memory_<<*τ*_growth_. However, both *τ*_aval_, which depends on the gravity conditions, and the duration of inclination ∆*t* can be varied. To derive theoretical solutions, we first assume that the curvature can be approximated by C≈*θ*_tip_/*L* as the initial response displays homogeneous curvature rates; (Chauvet *et al.*, 2016), and eqn (7) can be written at the tip with *s*=*L*:

$$\begin{array}{l} \frac{d\theta_{tip}}{dt}=-\frac{1}{\beta \tau_{growth}^{*}}G_{s}\left(L,t\right)\; \end{array}$$ (12)

with τ*_growth_ = *τ*_growth_*R*/*βL*. Different cases can be considered depending on the values of *τ*_aval_ and ∆*t* compared to *τ*_memory_ and τ*_growth_.

*1st case* τ_aval_ << *τ*_memory_ << τ*_growth_.

The avalanche is very rapid compared to the integration process and to the growth time, which from eqn (9) implies that for *s*=*L*: *A*_stato_(*L*,*t*) ≈ *A*_stem_(*L*,*t*) ≈ *θ*_incl_.

If ∆*t* >> *τ*_memory_, the integration process is fully saturated during the inclination, meaning from eqn (8) that *G*_s_(*L*,*t*) ≈ *βA*_stato_(*L*,*t*) ≈ *βθ*_incl_. From eqn (12) and the definition of $\tilde{\beta }$, it can be shown that in this case $\tilde{\beta }\approx \beta$, meaning that the gravisensitivity is independent of the duration of the inclination. In this case, we recover the classical ‘sine law’ used in the AC model.

If ∆*t* << *τ*_memory_, an estimate of the gravitropic signal is given by eqn (8): *G*_s_(*L*,*t*) ≈ (∆*t*/*τ*_memory_)*βθ*_incl_, which together with eqn (12) implies that $\tilde{\beta }\approx \beta (\Delta t/\tau_{\text{memory}})$.

*2nd case* τ_memory_ << *τ*_aval_ << τ*_growth_.

The avalanche is much slower than the integration process but faster than the bending of the plant. Equation (8) implies that *G*_s_ ≈ *βA*_stato_.

If ∆*t* >> *τ*_aval_, the statolith pile has time to relax to the horizontal and eqn (9) implies that *A*_stato_ ≈ *A*_stem_ ≈ *θ*_incl_ and again the gravitropic sensitivity defined by eqn (11) is given by $\tilde{\beta }\approx \beta$.

If ∆*t* << *τ*_aval_, eqn (9) gives an estimate of the statolith pile angle $A_{\text{stato}}\approx (\Delta t/\tau_{\text{aval}})\theta_{\text{incl}}$, implying that $\tilde{\beta }\approx \beta (\Delta t/\tau_{\text{aval}})$.

*3rd case* τ_memory_ << τ*_growth_ << *τ*_aval_.

The typical time of bending is small compared to the relaxation time of the statolith pile to the horizontal, implying that *A*_stato_ remains very small. At the tip, eqn (9) can be approximated by d*A*_stato_/d*t*(*L*,*t*) ≈ *A*_stem_(*L*,*t*)/*τ*_aval_ ≈ *θ*_tip_/*τ*_aval_. Because the integration process is very rapid, the gravitropic signal (eqn 8) is given by *G*_s_ ≈ *βA*_stato_. By differentiating eqn (12), it is found that the response of the tip angle with time obeys the following differential equation:

$$\begin{array}{l} \frac{d^{2}\theta_{tip}}{dt^{2}}\approx -\frac{1}{\tau_{growth}^{*}\tau_{aval}}\theta_{tip} \end{array}$$

Knowing that just after inclination, *θ*_tip_(*t*=0) = *θ*_init_ and d*θ*_tip_/d*t* = 0, a solution is

$$\begin{array}{l} \theta_{tip}\approx \theta_{incl}\cos\left(t\sqrt{\frac{1}{\tau_{growth}^{*}\tau_{aval}}}\right) \end{array}$$

This implies that

$$\begin{array}{l} \tau_{growth}^{*}\beta \frac{d\theta_{tip}}{dt}\approx \beta \sqrt{\frac{\tau_{growth}^{*}}{\tau_{aval}}}\sin\left(t\sqrt{\frac{1}{\tau_{growth}^{*}\tau_{aval}}}\right) \end{array}$$

Knowing that $\tilde{\beta }=\tau {*}_{growth}\beta {\left(\text{d}\theta_{\text{tip}}/\text{d}t\right)}_{\max}$, the following expressions are obtained for $\tilde{\beta }$:

if $\;\Delta \;t\ll \sqrt{\tau_{growth}^{*}\tau_{aval}}$, then $\tilde{\beta }\approx \beta (\Delta t/\tau_{\text{aval}})$.

If $\;\Delta \;t\gg \sqrt{\tau_{growth}^{*}\tau_{aval}}$, the time response of d*θ*_tip_/d*t* being sinusoidal, then the maximum is obtained when the sinus is equal to one. This implies that $\tilde{\beta }\approx \beta \left(\tau_{\text{growth}}^{*}\tau_{\text{aval}}\right)$.

From the different analytical limits derived in this section a phase diagram can be plotted showing the different regimes of the gravitropic response of a plant submitted to transient inclination (see below).

## Results

### The gravitropic response to transient inclinations follows a dose–response relationship

**Fig. 5.**
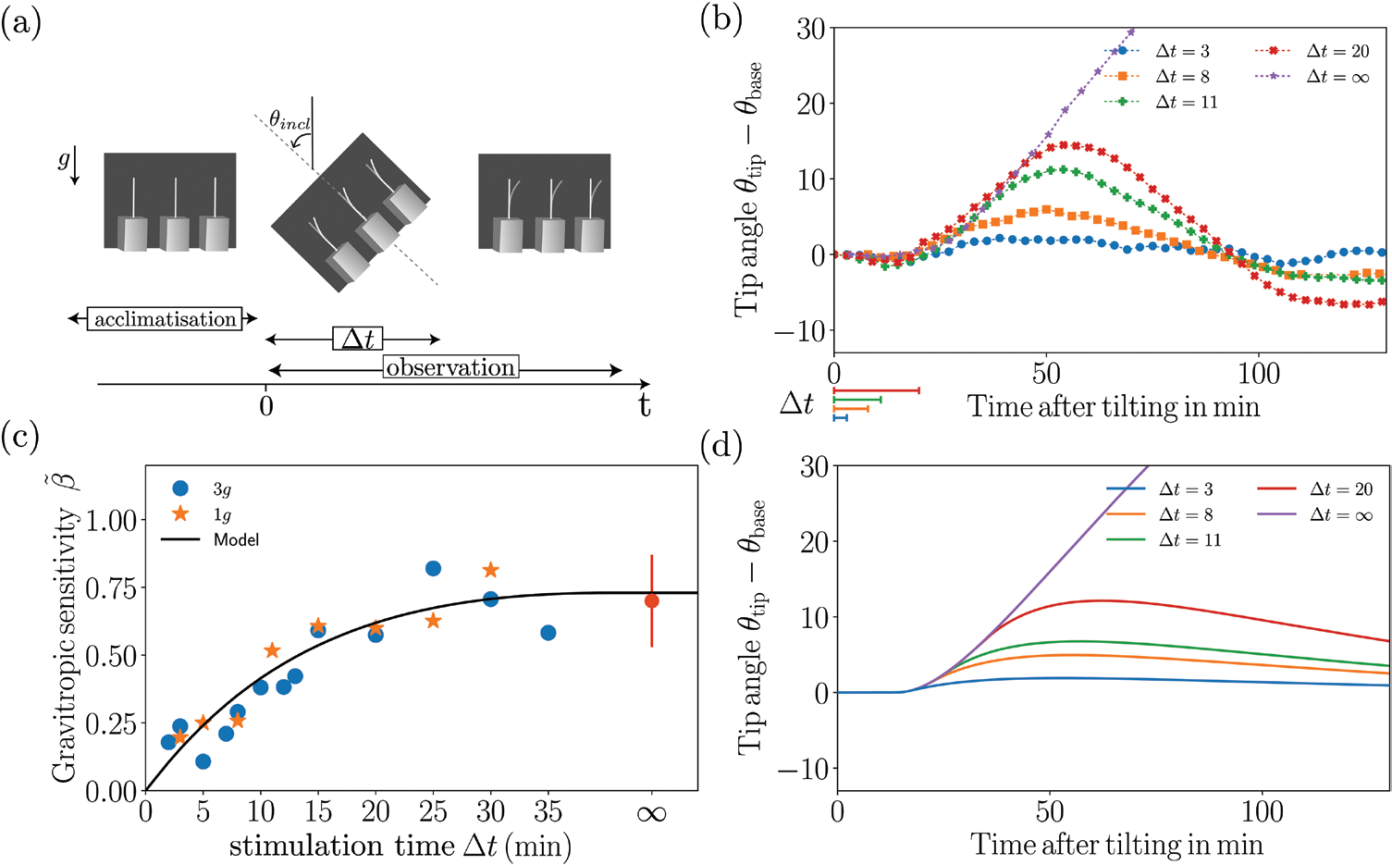
Plant responses to transient inclinations. (a) Diagram of a typical time sequence of an inclination applied to wheat coleoptiles. Note that in this diagram the gravity is vertical, i.e. corresponding to Earth gravity: it is inclined for *g*_eff_ = 3*g* obtained on the rotating table. (b) Time response of the tip angle *θ*_tip_ for various inclination durations (∆*t* = 3, 8, 11, and 20 min, indicated below the axis) under Earth gravity, *g*_eff_ = 1*g*. The time response for a permanent stimulus (∆*t* = ∞) is also shown. (c) The gravitropic sensitivity $\tilde{\beta }$ (eqn 11) as a function of the stimulation time ∆*t* for *g*_eff_ = 1*g* (stars) and *g*_eff_ = 3*g* (dots). The point at ∞ shows the mean value of $\tilde{\beta }$ for a permanent stimulus (from Chauvet *et al.*, 2016). (d) The predictions of the model for the same conditions as measured in (b).

We first present results concerning the responses of wheat coleoptiles subjected to transient inclinations at *θ*_incl_ = 45° over a time duration ∆*t* ranging from 2–35 min before being returned to the vertical (Fig. 5a). The response of *θ*_tip_(*t*)–*θ*_base_(*t*) averaged over the set of coleoptiles plotted for different values of ∆*t* at a gravity intensity *g*_eff_=1*g* (no rotation of the table) is shown in Fig. 5b. The inclination at the base *θ*_base_(*t*) is simply equal to zero before and after the inclination, and equal to *θ*_incl_ (45°) during the transient inclination. For each curve, the initial time *t*=0 corresponds to the beginning of the transient inclination, and the duration of the inclination is indicated on the graph by the segments below the axis. All the curves in Fig. 5b follow the same trend. First, after the inclination is imposed, there is always a delay of about 15 min before any bending motion is observed. After this delay, the tip angle increases, meaning that the plant bends in the direction opposite to that of the inclination. The tip angle then reaches a maximum before decreasing and returning to zero after a few oscillations. Increasing the duration of inclination ∆*t* increases the initial speed of bending and the maximum value reached by the tip angle.

The gravitropic sensitivity $\tilde{\beta }$ defined by eqn. (11) (Fig. 5c) first increased with the inclination duration ∆*t* before reaching a plateau, the value of which corresponded to $\tilde{\beta }$ measured when a permanent inclination was applied (∞ on the graph).

Interestingly, a gravitropic response was observed in our experiments as soon as ∆*t* was not zero, despite the reaction time. Therefore, no sensitivity threshold or minimum ‘presentation dose’ was observed in our system.

### The dose–response relationship is independent of the gravity intensity under Earth and hypergravity conditions

In microgravity, the gravitropic dose has been shown to be proportional to both the duration of stimulation ∆*t* and the gravity intensity. To check the validity of this law, experiments were carried out at a higher level of gravity intensity using a rotating table set-up (Fig. 1). In Fig. 5c the circles show the gravitropic sensitivity $\tilde{\beta }$ as a function of the inclination duration ∆*t* for an effective gravity *g*_eff_=3*g*. Surprisingly, data fall on the same curve as for *g*_eff_=1*g*. Therefore, in contrast with the dose–response reported in microgravity, no influence of the effective gravity on the gravitropic sensitivity was observed.

### Avalanche dynamics of the statolith piles in response to statocyte inclination

Our observation of a gravitropic response that was proportional to the stimulation time but independent of the gravity intensity questions the link between the dose–response relationship and the statolith dynamics proposed by Pouliquen *et al.* (2017) (see Introduction). Indeed, it would be expected that the motion of the statoliths should depend on the gravity intensity. To address this issue, we have observed the motion of statoliths in wheat coleoptile statocytes in response to inclination (see Figs 2 and 3).

**Fig. 6.**
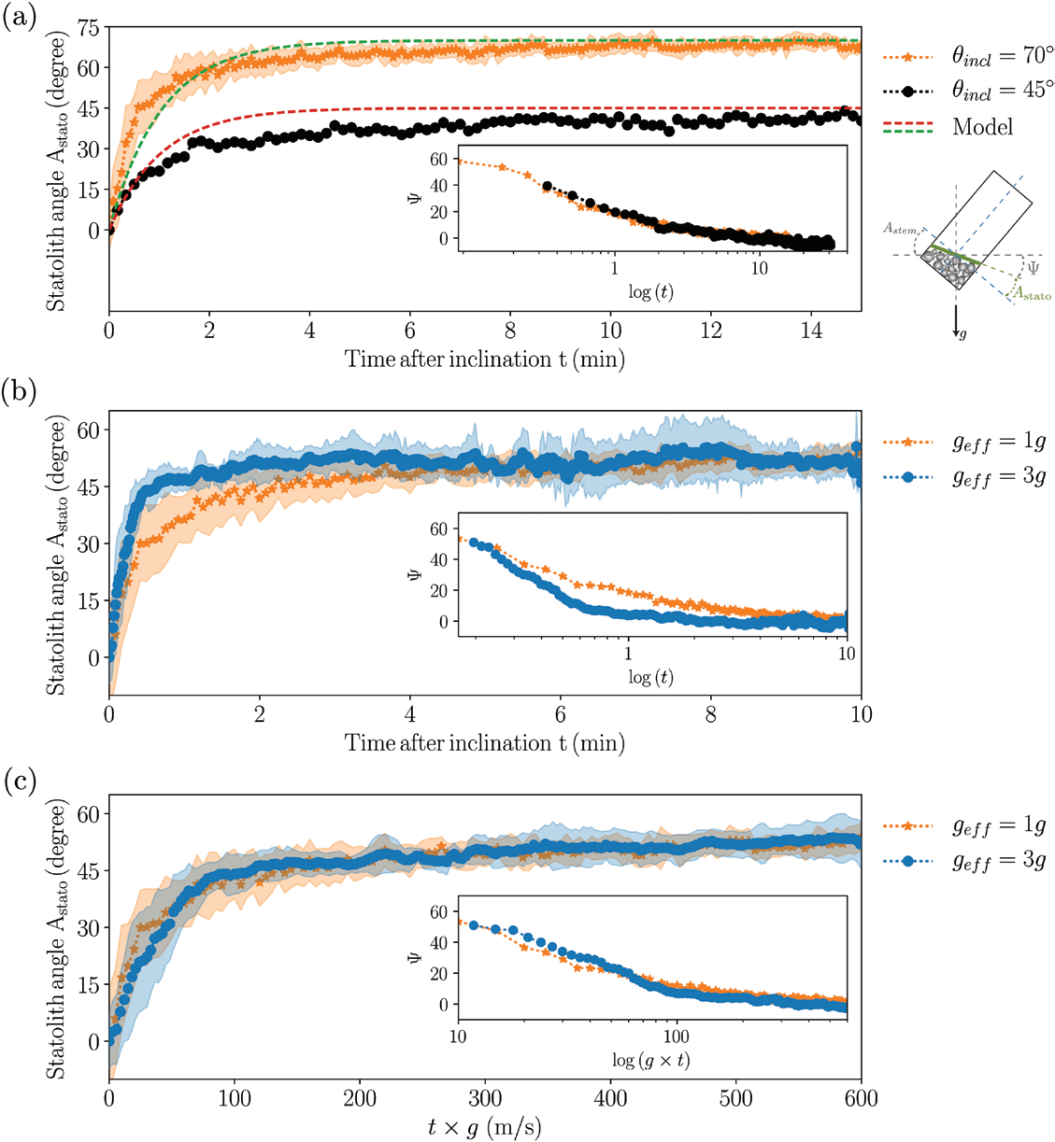
Avalanche dynamics of statoliths in wheat coleoptiles. (a) Relaxation with time of the statolith angle *A*_stato_ for two initial inclinations of 70° and 45°. The dashed lines show the predictions of the theoretical model. Inset: time response on a logarithmic scale of the angle of inclination of the pile from the horizontal, *Ψ*(*t*) (inclination of the pile from the horizontal). (b) *A*_stato_(*t*) for two different gravity intensities, *g*_eff_ = 1*g* and *g*_eff_ = 3*g*. (c) *A*_stato_ as a function of *g*_eff_ × *t* for *g*_eff_ = 1*g* and *g*_eff_ = 3*g*. Shaded areas correspond to ±standard deviation.

The time evolution of the statolith angle *A*_stato_ (defined as the angle between the free surface of the statolith pile and the small side of the cell) averaged over several cells (typically 10) for an initial inclination of the sample of either 70° or 45° are shown in Fig. 6a. As soon as the inclination was imposed, the statoliths started to flow in a coherent manner along the cell wall, with no noticeable delay. *A*_stato_ increased within few minutes from zero to its final value equal to *A*_stem_, the inclination angle imposed to the system, meaning that the free surface of the statolith pile relaxed to the horizontal (Fig. 6a). A more careful analysis revealed that these dynamics of relaxation were characterized by two regimes (Bérut *et al.*, 2018). First, the free surface rapidly moved and reached an angle of about Ψ=10° from the horizontal in about 2 min (Fig. 6a inset); the free surface of the statolith pile then crept slowly and came back to the horizontal in about 20 min with logarithmic dynamics. Interestingly, the two dynamics obtained for the two different initial inclinations (70° and 45°) could be superimposed if the 45° curve was shifted so that its initial time coincided with the time when the avalanche at 70° reached 45° (Fig. 6a inset). This suggests that the dynamics of the statolith avalanches are controlled by the viscous drag of the surrounding cytoplasm rather than by inertial effects. This is also consistent with the low value of the Reynolds number for this flow: *Re* = *ρUL*/*η* ≈ 10^–6^, where *ρ* is the density of the cytoplasm (≈10^3^ kg m^–3^), *U* is the maximal statolith avalanche velocity (≈10 µm min^–1^), *L* is the statolith pile size (≈20 µm), and *η* is the cytoplasm viscosity (≈10 mPa-s) (Bérut *et al.*, 2018).

### The time-scale of the statolith avalanche is controlled by the gravity intensity

In order to investigate the influence of the gravity intensity on the avalanching process, cell preparations were observed using a specialized set-up (see Methods). The avalanche dynamics at *g*_eff_=1*g* and *g*_eff_=3*g* are compared in Fig. 6b for the same initial inclination relative to the direction of the effective gravity vector. The dynamics of both were characterized by an initial rapid avalanche regime followed by a slow creeping motion, with the transition being sharper at 3*g* than at 1*g*. The main difference was apparent in the time scale. The avalanche at higher gravity intensity was faster, as expected from physical arguments (see ‘Statolith avalanche’ in the Methods).

According to eqn (4), the avalanche duration *τ*_aval_ should be three times longer at *g*_eff_=1*g* than at *g*_eff_=3*g*. To confirm this prediction, we plotted the change of the pile angle, Ψ, as a function of *g*_eff_×*t* (Fig. 6c). The two curves corresponded, thus showing that the typical time-scale of the avalanche dynamics is inversely proportional to the gravity intensity: *τ*_aval_ ∝ 1/*g*_eff_.

### Evidence of a memory process independent of the dynamics of the statoliths

**Fig. 7.**
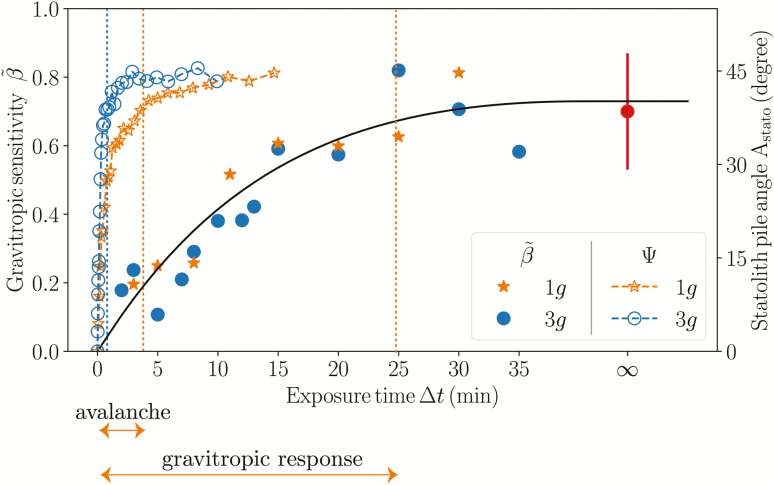
Comparison between the avalanche dynamics of the wheat coleoptile statoliths (open symbols) and the gravitropic response to transient inclinations (closed symbols), showing that the avalanche is much too rapid to explain the increase of the response with ∆*t*.

The time response of the statolith avalanche at the cellular level and the gravitropic dose–response at the whole-plant level can now be compared on the same plot (Fig. 7). For both gravity conditions *g*_eff_=1*g* and *g*_eff_=3*g*, the avalanche time appeared to be much shorter than the typical response time of the dose curve. This meant that for all the transient inclinations investigated (∆*t* between 2–20 min), the statolith piles had time to reach their final position well before the end of the stimulus (*τ*_aval_ < ∆*t* for all experiments). The increase of the gravitropic response with the stimulation time reported here therefore cannot be attributed to a limited excursion of the statoliths during ∆*t*, as suggested by Pouliquen *et al.* (2017). This suggests that the observed dose-like behaviour was due to another limiting process along the signalling pathway that integrates the initial signal induced by displacement of the statoliths, thus providing the system with a memory (Baldwin *et al.*, 2013).

### A mathematical model based on a hierarchy of time-scales predicts the dose–response relationship

Our experiments both at the plant scale and at the cellular level showed that the gravitropic response to various transient inclinations and gravity levels was controlled by at least four different time scales. The longest is set by the growth rate of the plant and is given by $\tau_{\text{growth}}=1/\dot{\varepsilon }$ (~1200 min; see Methods). The shortest time-scale is the statolith avalanche duration, *τ*_aval_, which was ~2 min, as deduced from our *in situ* observations of the statolith dynamics. However, the analysis of the response to transient inclinations highlighted two other time scales. First, a reaction time scale *τ*_reaction_ (or delay) of about 15 min between the first stimulation and the first bending response, during which no bending was observed. Second, a new memory time scale *τ*_memory_ of about 20 min, independent of the statolith dynamics, as evidenced from the gravitropic dose–response relationship that was observed during transient inclination. From this set of information, we constructed a mathematical model that we term A-SD-M (angle-statolith dynamic-memory) to predict the bending response of shoots to arbitrary gravitropic stimulations (see eqns 7–10 in the Methods). It extends previous kinematic models of gravitropism (e.g. Bastien *et al.*, 2014) by taking into account the coupling between (i) the statolith dynamics at the cellular level controlled by the avalanche time, *τ*_aval_, (ii) the gravitropic signal obtained from integration of the position of the statoliths during the time *τ*_memory_ with a delay *τ*_reaction_, and (iii) the change of curvature of the shoot in response to the gravitropic signal and controlled by the growth time, *τ*_growth_ (see Methods for full details). We focus on the early bending period that is dominated by the gravitropic response, and other processes such as proprioception have been omitted.

The predictions of the model for *g*_eff_=1*g* are presented in Fig. 5d, in which the time response of the tip angle is plotted for a transient inclination at 45° and various stimulation periods ∆*t* that correspond to the experiments (Fig. 5b). During the first phase of the process, the model predictions quantitatively matched the experiments, with the same initial resting time controlled by *τ*_reaction_ followed by an increase of the tip angle up to a maximum. The second phase, when the tip angle decreased and the plant returned to vertical, was slower in the model than in the experiment, but for this long-term response it is likely that proprioception was no longer negligible (Bastien *et al.*, 2014), thus requiring additional terms to be added to the model. Here, we focus on the gravitropic sensitivity $\tilde{\beta }$, calculated from eqn (11) in which d*θ*_tip_/d*t*, the maximum slope of the tip angle as a function of time, is estimated from Fig. 5d. The prediction of the model for various stimulation periods ∆*t* is given in Fig. 5c (continuous line) together with the experimental measurements (symbols). The model fits well to the experimental data and reproduces both the increase and saturation of the response when the inclination duration ∆*t* is larger than the memory time *τ*_memory_.

### A phase diagram to unify the gravitropic response under hypo- and hypergravity conditions

The A-SD-M model thus predicted the gravity-independent dose–response relationship observed in our experiments under Earth and hypergravity conditions. An important question is whether the same model can also predict and explain the gravity-*dependent* dose–response that is observed for hypogravity conditions (Perbal and Driss-Ecole, 1994; Brown *et al.*, 1995; Driss-Ecole *et al.*, 2008). In the model, the gravity intensity comes into play only in the avalanche time, *τ*_aval_, which is proportional to 1/*g*_eff_ (the three other time-scales, *τ*_growth_, *τ*_memory_, and *τ*_reaction_, are assumed to be controlled by biochemical processes independent of gravity). Decreasing gravity is then equivalent in the model to increasing the avalanche time scale. To understand the model prediction in hypogravity conditions, we systematically varied the avalanche time in the simulations. For each value of *τ*_aval_, the inclination period ∆*t* can be varied, and a phase diagram can be constructed in the plane (*τ*_aval_, ∆*t*) showing the different gravitropic response laws predicted (see Methods for calculation details).

**Fig. 8.**
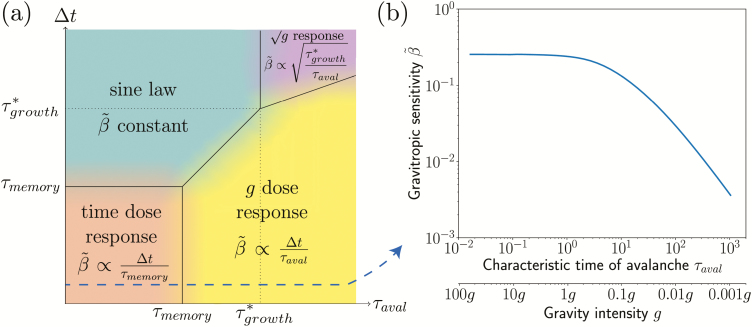
(a) Phase diagram of the gravitropic sensitivity $\tilde{\beta }$ computed from the A-SD-M theoretical model (logarithmic scales). Different regimes are observed depending on the values of the avalanche time *τ*_aval_ and of the inclination time ∆*t* relative to the memory time *τ*_memory_ and the modified growth time $\tau {*}_{\text{growth}}$ (see Results). (b) Gravitropic sensitivity $\tilde{\beta }$ as a function of the avalanche time *τ*_aval_ (and the equivalent gravity intensity *g*_eff_, given that *τ*_aval_ ∝ 1/*g*_eff_), for an inclination time ∆*t* = 5 min (<*τ*_memory_). The other constants (i.e. values of the parameters in the A-SD-M model) were taken from the experimental calibration for wheat coleoptiles: *τ*_memory_ = 15 min and *τ*_growth_ = 1200 min.

The phase diagram (Fig. 8) revealed the existence of four different regimes depending on the relative values of *τ*_aval_ and ∆*t* compared to *τ*_memory_ and *τ*_growth_ (*τ*_reaction_ plays a minor role in the gravitropic sensitivity as its influence is only to delay the response). The first regime was obtained when both *τ*_aval_ and the stimulation period ∆*t* were smaller than *τ*_memory_ (orange zone in Fig. 8). Within this range of parameters, a dose–response relationship independent of the gravity intensity was predicted by the model, with $\tilde{\beta }\propto \Delta t/\tau_{\text{memory}}$. This regime typically corresponds to the experimental conditions of our current study, i.e. transient tilting experiments in Earth or hypergravity conditions. The second regime was obtained when $\tau_{\text{aval}}<\tau_{\text{growth}}$ and the stimulation period $\Delta t>\text{Max}\left|\tau_{\text{memory}},\tau_{\text{aval}}\right|$ (blue region). In this regime, the inclination stimulus can be considered as permanent. The integration process is fully saturated and the statolith avalanche motion is finished, implying that the gravitropic response depends on neither the inclination period ∆*t* nor the gravity, but instead only depends on the inclination angle. This regime typically corresponds to the sine law response to steady inclination as studied by Chauvet *et al.* (2016). The third regime was observed when the avalanche time *τ*_aval_ was longer than both the memory time *τ*_memory_ and the stimulation period ∆*t* (yellow region). During the stimulation period, the integration process is saturated but the movement of the statoliths is not yet completed (Pouliquen *et al.*, 2017). In this case, the gravitropic response is also a dose–response relationship, but now the response depends on the gravity intensity: $\tilde{\beta }\propto \Delta t/\tau_{\text{aval}}\propto g_{\text{eff}}/\Delta t$. This regime is compatible with experiments in low-gravity conditions, where a *g*_eff_-dependent dose–response relationship has been observed (for the wheat coleoptiles studied here, $\tau_{\text{aval}}>\tau_{\text{memory}}$ means that *g*_eff_ < 0.1*g*). The fourth regime predicted by the model corresponded to $\tau_{\text{aval}}>\tau_{\text{growth}}$ and $\Delta t>\sqrt{\left(\tau_{\text{aval}}\;\tau_{\text{growth}}\right)}$ (purple region). In this case, the gravitropic response is independent of ∆*t* but depends on the gravity intensity $\tilde{\beta }\propto \sqrt{\left(\tau_{\text{growth}}\backslash \tau_{\text{aval}}\right)}\propto \sqrt{g_{\text{eff}}}$ This last regime, which is controlled by the growth time and observed at very low levels of gravity and for long stimulation periods, has, to our knowledge, never been explored (for the wheat coleoptiles studied here, *τ*_aval_ > *τ*_growth_ means *g*_eff_ < 10^–3^*g*).

The A-SD-M model therefore enables to unify the three main kinds of gravitropic responses reported here and in the literature: (1) the gravity-independent dose–response relationship observed here for transient stimuli under Earth and hypergravity conditions; (2) the gravity-independent sine law observed for very long or steady stimuli reported as by Chauvet *et al.* (2016); and (3) the classical gravity-dependent dose response discussed in the microgravity literature. In particular, for short stimuli (∆*t*<*τ*_memory_), the model predicts that the transition between a ∆*t* dose–response and a *g*_eff_∆*t* dose–response relationship occurs when the statolith avalanche time is increased, i.e. when the gravity intensity is decreased. This transition is illustrated in Fig. 8b, where the sensitivity is independent of *g*_eff_ when it is high and decreases linearly when it is low. The transition occurs when the avalanche time becomes longer than the memory time, i.e. when the slowest process in the signalling pathway is no longer the memory process and instead becomes the dynamics of the statolith avalanche.

## Discussion and conclusions

Despite much progress in recent years (Strohm *et al.*, 2012; Baldwin *et al.*, 2013; Toyota and Gilroy, 2013; Pouliquen *et al.*, 2017; Taniguchi *et al.*, 2017), the spatio-temporal sequence of events that relates the early detection of gravity by the statoliths in the statocyte cells to the macroscopic gravitropic response of the plant, as well as to the molecular factors involved, is still not well understood. In this study on wheat coleoptiles, we used a combination of plant-scale experiments and live-cell imaging techniques on rotating devices in order to investigate both the kinematics of shoot bending induced by transient inclinations and the dynamics of the statoliths inside the statocytes during the inclination for different levels of gravity intensity. We then rationalized our experimental results into a new mathematical model, the A-SD-M model, that takes into account the statolith avalanche dynamics, a reaction time, the tropic growth process, and a novel memory-integration process. Once calibrated, the A-SD-M model quantitatively reproduced the experimental measurements of the response to transient inclination of the coleoptiles under both Earth and hypergravity conditions.

We developed a framework for predicting the early bending response of shoots to arbitrary gravistimulation, based on the time-scale of the new memory-integration process and on the time-scale of the movement (avalanche) of the statoliths. This framework can be represented as a phase diagram of the different gravisensing regimes predicted by the A-SD-M model as a function of the intensity and duration of the stimulus (Fig. 8a). This phase diagram enables us to unify within the same framework the gravitropic response to unsteady (dose–response) and steady (sine law) stimulations, from microgravity to hypergravity conditions.

Our study thereby enables to reconcile two classical laws found in the plant gravitropism literature, namely the gravity-independent sine law in response to steady inclinations and the gravity-dependent dose–response relationship reported for transient stimulations in microgravity. The key ingredient lies in the existence of an intrinsic time-scale in the gravitropic signalling pathway that filters the temporal variations of the imposed stimulation. Previously, we hypothetized that this intrinsic time-scale could be set by that of the statolith avalanche, which varies in inverse proportion to the gravity intensity (Pouliquen *et al.*, 2017). However, our novel experiments under Earth and hypergravity conditions partially falsified this explanation. In addition, these experiments revealed a new gravity-independent dose–response relationship with a time scale *τ*_memory_ that was much longer than that of the statolith avalanche, indicating the existence of a more complex scenario. We therefore had to introduce a novel hypothesis that involves a memory process that integrates the initial signal induced by statolith displacement. Under Earth and hypergravity conditions, the integrative-memory process is dominant, whereas it disappears as the gravity intensity becomes very small and the time for avalanche completion *τ*_aval_ becomes larger than *τ*_memory_ (Fig. 8a).

Overall, our study highlights the importance of the time-scale hierarchy, from statolith motion to differential growth-induced bending, to predict the response of plants to arbitrary gravitropic stimulations. Key assumptions in the model are the introduction of the memory time-scale *τ*_memory_, which buffers the initial signal induced by statolith displacement, and the introduction of a reaction time-scale *τ*_reaction_, which postpones the response. Our results show the two processes to be independent of the gravity intensity and thus they cannot be attributed to the dynamics of the statoliths. Therefore, their origin has to be located in the downstream molecular signalling pathway.

The existence of a delay, or reaction time *τ*_reaction_ has previously been discussed in the literature, but only rarely. Iino *et al.* (1996) found a reaction time for rice coleoptiles of ~30 min, similar to that estimated for maize roots by Nelson and Evans (1986). This is longer than our estimate of *τ*_reaction_ = 13 min found in our current study, but it remains in the same order of magnitude (as our estimate was the minimum time before a response was observed and not the mean time). The underlying process behind the reaction time has not yet been determined. Our study shows that it should not affect the amplitude of the signal but simply postpone it through its propagation. Auxin transport and the typical time for the onset of the lateral gradient could be a candidate for this delay. Philippar *et al.* (1999) reported a delay of ~10 min for the gradient to be created in coleoptiles, which is close to our measured *τ*_reaction_. An alternative explanation could be a mechanosensitive transient inhibition of the elongation growth when the coleoptile bends under its own weight after tilting. The mechanosensing of applied bending strains has been shown to transiently stop elongation growth, a response that belongs to the thigmomorphogenetic syndrome (Moulia *et al.*, 2015). However, our experiments with simultaneous recording of the elongation growth and the gravitropic response tend to rule out this explanation (Fig. 2; see Chauvet *et al.*, 2016). A final candidate mechanism, and probably the most likely, comes from accounting for the time for the events downstream of the auxin signalling to take place in the elongation cells. Whatever the origin of this reaction time, its importance to the overall response dynamics should not be overstated. Indeed, *τ*_reaction_ only plays a minor role in the gravitropic sensitivity as it simply introduces a constant time delay in the response.

Of more importance is our demonstration of an integration process associated with *τ*_memory_, and independent of gravity, which filters the signal induced by the statoliths. The relocation of auxin efflux carriers (PIN proteins) could be a potential candidate for this process. Some of these PINs, (PIN3, PIN4, and PIN7) are involved in the gravisensing response, as shown by studies on mutants (Friml *et al.*, 2002; Rakusová *et al.*, 2011). Observations using a fluorescent reporter of PIN3 have shown that its repartitioning along the cell membrane in roots and in shoots changes rapidly after statolith displacement (Friml *et al.*, 2002; Rakusová *et al.*, 2011; Baldwin *et al.*, 2013). The resulting asymmetric distribution of fluorescent PIN3 appears few minutes (~2 min) after gravistimulation and continues to evolve for more than 20 min (Friml *et al.*, 2002), as shown by the augmentation of the concentration of endosomes containing PIN3 in roots (Kleine-Vehn *et al.*, 2010). The time-scale of the relocalization is thus compatible with our observations in the dose–response curve, meaning that this cumulative process could be a good candidate for the underlying mechanism. Should this be the case, our study would reveal a novel function for PIN3 relocalization, namely its capacity to filter and buffer transient and fluctuating stimuli (such as those provided by large gusts of wind) through integration. However, a better quantification of the temporal dynamics of the PIN relocation processes using live-cell imaging methods would be necessary in order to reach a firm conclusion.

Finally, our study contributes to predictive approaches in computational biology. First, our approach can be extended to mutants or transgenics plants. For example, studying starchless mutants would be of interest to explore the phase diagram predicted by our theory. With the motion of the statoliths being slower in these mutants due to a lower density, the avalanche time may become higher than the memory time-scale under Earth gravity, thus mimicking microgravity conditions. Second, from a modelling point of view, our mathematical model could be extended to include the proprioception (following Bastien *et al.*, 2013, 2014) and hence to provide an integrative model of gravitropic control with a direct phenomenological cellular basis for the sensory mechanisms. But much still remains to be determined about the sensors and the signalling pathway involved in plant proprioception.

## Data deposition

Raw data from the experiments are available on the open-source database Zenodo. http://dx.doi.org/10.5281/zenodo.1404150
